# Interval Kicking Program for the Punting and Place-Kicking Athlete: A Systematic Literature Review and Need Analysis

**DOI:** 10.7759/cureus.19725

**Published:** 2021-11-18

**Authors:** Austin G Wynn, Andrew P Collins, Elizabeth Nguyen, Eric Sales, Harrison Youmans, Daryl C Osbahr, Ibrahim Zeini, Michelle Henne

**Affiliations:** 1 College of Medicine, University of Central Florida, Orlando, USA; 2 Orthopedics and Sports Medicine Group, Orlando Health, Orlando, USA; 3 Rothman Orthopedic Institute Florida, AdventHealth Orlando, Orlando, USA; 4 Orthopedic Institute, AdventHealth Orlando, Orlando, USA; 5 Sports Medicine, Releve Sports Medicine, Winter Haven, USA

**Keywords:** rehabilitation, football, rugby, interval program, lower extremity

## Abstract

Interval programs have been developed for multiple sports, allowing athletes to return to sport-specific activity in a graded fashion, minimizing the risk of reinjury. However, there currently exists a gap in the literature surrounding the use of interval programs for the rehabilitation of punting and place-kicking athletes. We aim to perform a systematic review of the literature examining the use of interval kicking programs to aid punting and place-kicking athletes following a lower-extremity injury. Following PRISMA guidelines, a review was performed using PubMed and MEDLINE databases to evaluate the literature surrounding interval kicking programs for punting and place-kicking athletes. Search terms were combined using Boolean operators of “AND” and “OR”. Articles included in this review met these criteria: 1) included patients with lower-extremity pain/injury, 2) reported a return to sport progressive program, and 3) analyzed the measure’s ability to predict a successful return to sport. The initial search returned 115 articles. Seventy-nine of these articles were excluded after initial screening, leaving 36 full-text articles for final review. Of these final articles, there were no studies outlining the use of interval kicking programs by punting or place-kicking athletes. Of the articles reviewed, the most relevant was an interval kicking program developed by Arundale et al. specifically for the soccer athlete. Punting and place-kicking use biomechanically distinct patterns of movement, warranting a specific interval program. This review identified a gap in knowledge surrounding the use of interval programs in the rehabilitation of punting and place-kicking athletes. This review will now describe what is currently known regarding biomechanics of punting and place kicking, the injuries experienced by these athletes, and the benefit an individualized interval program could provide. There currently exists a gap in the literature surrounding the use of interval programs for the rehabilitation of punting and place-kicking athletes. The biomechanics and application of these skills are distinct, and an interval program designed specifically for these athletes is warranted. Future research should be dedicated to the development, implementation, and analysis of an interval kicking program designed for these athletes.

## Introduction and background

Punting and place kicking are a part of regular sport-specific activity within American football, rugby, and Australian rules football. Injuries to American kickers, and rugby and Australian rules football players predominantly affect the lower limb [1-3]. After any injury, the main goal is a safe return to sport activity and to ensure that an athlete’s rehabilitation will reduce the risk for reinjury. To this end, progressive interval programs have been developed for multiple sports that allow athletes to return to sport-specific activity in a graded fashion, ensuring that the risk of reinjury is minimized. These programs have previously been developed for use following upper-extremity injury for baseball, tennis, and golf, as well as other overhead throwing sports [4-6]. A return to kicking protocol was developed by Arundale et al. to be used for soccer players following a lower-extremity injury [7]. The kicking mechanics of the punter or place kicker in American football, rugby, and Australian rules football differ from soccer, including the use of a “tee” in place kicking and the use of the forefoot as a contact point in punting instead of the instep kick commonly utilized in soccer [8-10]. Of note, goal keepers in soccer use a punting motion similar to the other mentioned sports but no mention of programming specific to goal keepers was made in the Arundale article. To the best of the authors’ knowledge, there is currently a gap in the literature regarding interval-kicking programs for the punter or place kicker. This review aims to establish what the literature currently states regarding interval programs in relation to the punter and placekicker as well as the need for future development of a program that meets the specific needs of these players. 

## Review

**Figure 1:**
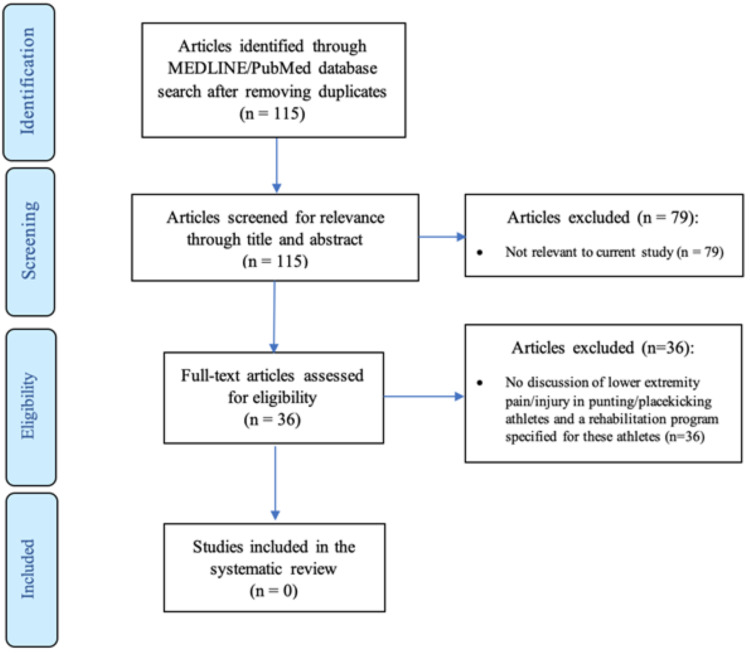
Systematic review article screening flowchart

A systematic review of the literature evaluating the presence and use of interval programs in regard to punting and place kicking was performed using the PRISMA guidelines [11]. A search was performed in the PubMed and MEDLINE databases in January 2019 using the terms outlined in Table 1 and all literature available at the time were included in the review. Terms were combined using Boolean operators with individual terms in each column being combined with “OR” and with “AND”. After the search, articles were screened for relevance by title and abstract. Articles were excluded based on the following criteria: there was no mention of American football/Australian rules football/rugby, the article was a literature review, there was no discussion of lower-extremity injury, the full text was not available to the author's institution, or the article was not relevant to the current question. All full-text articles were reviewed independently by at least three of the authors to determine their relevance for inclusion in the review. Any article that was in question was discussed among the authors until a majority consensus was reached. A flow chart of this process can be seen in Figure 1. 

**Table 1:** Search terms used in PubMed and MEDLINE databases Search terms that mapped to specific MeSH terms are italicized.

“Kicking”	"American football"	“Lower extremity injury”
“return to kicking”	“football”	“rehabilitation”
“interval kicking program”	“rugby”	“progression”
“interval program”	“Australian rules football”	“hip flexion”
“kicking interval”		“leg”
“punter”		“lower extremity kinematics”
“placekicker”		“lower extremity injury”
“kicker”		“biomechanics”
“kick”		“return to play”
		“recovery of function”
		“return to sports”
		“muscle forces”
		“obturator internus”
		“obturator externus”
		“quadratus femoris”
		“adductor”
		“hamstring”
		“rectus femoris”

Results

The search was performed in February 2020 and returned an initial 115 articles. After an initial screening by abstract and title for relevance to the research question, 36 articles remained for consideration. After final review, no article could be found that presented an interval program that was tailored for the punting and kicking athlete in a non-soccer sport. The most relevant article was the interval kicking program that was published by Arundale et al.; however, as previously stated, this program was developed for the soccer athlete. The search mainly returned articles that describe the epidemiology of injuries in American football, rugby, and Australian rules football as well as the kinematics of punting and place kicking. As the mechanics of punting and place kicking are different in football or rugby than kicking in soccer and given that in-game applications of kicking are unique to each of these sports, a specific interval program for these athletes is needed. The remainder of this article will describe what is currently known regarding kicking mechanics of punting and place kicking, the injuries experienced by these athletes, and further elaboration on the benefit that an individualized interval program could provide. 

Currently, the lack of a return-to-kicking program specific for American football athletes could reduce optimal recovery of players, increasing the burden of injury faced by both the individual player and the team. Interval programs that focus on injury-specific training, plyometrics, and neuromuscular control drills can enhance player recovery and reduce days of play lost [6,7]. Many of these interval programs are designed to be performed in conjunction with other rehabilitation training, and stability of the injured limb should be assessed before beginning the program to reduce risk of additional injury or reinjury. This readiness for return to kicking and to play should be measured objectively with normative values to ensure an informed, unbiased decision concerning the athlete’s readiness [12,13]. Similar to when assessing the performance of a baseball pitcher, the radar gun can be used to assess the player’s ability to contact the ball and whether or not pain or discomfort occurred. A tool for measurement of ground reaction forces may also be beneficial. While analyzing joint and limb stability, some other measures such as kicking distance, height, and accuracy are all objective analyses that can be used as comparison to the pre-injury kicker’s values to substantiate a return-to-play determination for the athlete.

Injuries to the punting and place-kicking athlete in American football, Australian rules football, and rugby

In the National Football League (NFL), the population of professional kickers experiences injuries at a rate of 24 per year, while the total number of kickers in the position was only 45 in the 2020 team rosters [1,14]. This injury rate translates to over half of the NFL kickers experiencing injuries of varying detriment per season. With only 32 teams in the NFL, this injury rate could exclude some teams from having a reliable kicker, depending on the severity of the injury. These injuries can lead to significant medical costs for the teams, decreased overall team performance, and loss in playing time [15]. Over a 20-year surveillance period, it was shown that over 89% of both place kickers and punters in the NFL were right-footed, and the majority of injuries in the kickers were overrepresented for the dominant leg [1]. The vast majority of NFL kicker injuries are acute or traumatic, and nearly all of the most common diagnoses reported involve the lower extremities, barring concussion [1]. In 2015, the players of the Australian Football League, a sport that utilizes similar kicking mechanics to American football, have shown 41.7 injuries per club per season, just under one injury per player [3]. Furthermore, the kicking action used in Australian rules football is considered to be responsible for many lower-limb injuries [16]. These injuries in the AFL were consistent over time, ranging from 37.6 to 48.1 injuries per season [3]. In each of these professional leagues, lower-limb injuries are common occurrences, and several factors affect return to play following the injury, including injury type, severity, location, and rehabilitation protocol.

Studies have shown that NFL kicker injury occurs at significantly higher rates during games (17.7 per 1000) than during practice (1.91 per 1000). Australian Rugby League (ARL) players experience a similar pattern of increased injury incidence during matches than in practice. Many of the injuries experienced during games of NFL kickers and ARL players are non-contact mechanisms [1,17]. American football kicking athletes have a lower injury incidence than all other players, likely due to their decreased volume of position players in combination with the decreased frequency of direct contact in their position [7].

In professional American football, muscle strains account for over 70% of kicker-related injuries [1]. Most muscle strains occur due to eccentric loading, which is compounded in kicking sports when the foot strikes the ball [18-21]. Additionally, this risk of muscle strain injury and time to return to play following injury both increase with player age [1]. Injuries to the thigh region are the most common, as adductor, hamstring, and quadriceps muscle strains account for the three most common leg-related injuries in kickers [1]. While adductor and quadriceps strains are much more common in the right side (83% and 80% right-sided, respectively), hamstring strains are only 50% right-sided, indicating that the planted limb is also at risk for hamstring strains [1]. Additionally, hamstring injuries represent the most common non-contact injury in American football as a whole, as well as rugby, Australian rules football, and soccer [22]. Hamstring injuries also exhibit high rates of recurrence with 32% of hamstring injuries in American football being attributed to previous hamstring injuries. Similar rates of 23% and 21% are seen in Australian rules football and rugby union, respectively [22-25]. Also notable is that reinjury tends to be more severe, compounding the time it takes to return to play [26-29]. Furthermore, hamstring injuries in American football have been associated with decreased performance when returning to play [29,30]. From the 20-year NFL kicker injury surveillance period, patterns of injury occurrence vary by muscle group, sidedness, athlete age, and average days of play lost. Of the most common NFL kicker injuries, quadriceps muscle strains led the average days of play lost (36 days), while the second most debilitating injuries were groin strains and medial collateral ligament sprains (each averaged 20 days of play lost) [1].

In the same group, ligament sprains make up a smaller portion of the total group’s injuries, accounting for approximately 18% of all injuries of NFL kickers [1]. The majority of these ligament sprains occur at the lumbosacral ligament (56.8% of sprains), while ankle sprains constitute a lesser portion of sprains (anterior talofibular ligament accounts for 23.5% of all ligamentous sprains), and medial collateral ligament sprains slightly less than that (19.6% of all ligamentous sprains) [1]. The anterior talofibular ligament sprains disproportionately affected the left leg, indicating that it has a high risk for injury of the planted ankle while kicking [1]. The high rate of lumbosacral ligament sprains seems unique to American football, whereas similar injuries in Australian football, Rugby, and European football are much less prevalent [2,3,31-33]. In American football, medial collateral ligament sprains are the most common knee ligamentous injury, similar to professional European footballers [1,33,34]. In American kicking athletes, ligament sprain type and severity altered the average days of play lost; lumbosacral, anterior talofibular, and medial collateral ligament sprains resulted in an average of 16, 9, and 20 days of play lost, respectively [1]. Punters suffered a disproportionately higher percentage of lateral ankle sprains and lumbosacral injuries when compared with placekickers [1].

The leading anatomic areas of injury in descending order for the American football kicking athletes were the pelvis/hip, thigh, and, finally, knee, while the leading types of injury were muscle-tendon followed by ligament/joint [1]. Additionally, when comparing punting and place-kicking injuries in NFL kicking athletes, 47% of injuries occurred during punting, while 53% occurred during place kicking. Comparing injuries in each of these activities displayed no statistically significant differences in mean days lost to injury, setting of injury (practice versus game), injury mechanism, laterality of injury, or likelihood of necessity for surgical treatment.

Punting and place-kicking biomechanics

Kicking kinematics require sufficient range of motion through the movement, muscle strength, and neuromuscular control, all of which can be hindered by a lower-extremity injury. The planted foot during the kicking movement is of equal importance to the striking foot, as a balanced and stable base is necessary to develop force and accuracy when kicking [35,36]. Kicking in American football, rugby, and Australian rules football takes on two kinematically distinct forms, the punt and the place kick. The punt kick is seen in many sports including rugby, American football, Australian rules football, and by the goalkeeper in soccer [8]. This type of kick can be used to advance the ball in rugby, Australian rules football, or soccer, as well as a means to return the ball to the opposing team if the team in possession of the ball fails to meet the line to gain within a series of downs [8,37]. The motion of punting can be broken up into six distinct phases with individual kinematic characteristics, which are run-up/approach, backswing, wind-up, forward swing, follow through, and recovery [8].

The punt kick starts with a six-to-eight-step approach and ends with a strong concentric contraction of the kicking leg hamstring to begin the backswing. During this phase, the ball is dropped and the upper extremity contralateral to the kicking leg is abducted to provide balance during the kick. The backswing is characterized by full extension of the hip of the kicking leg as well as increased activity of the kicking hamstring and increased activity of the gluteal muscles in the support leg. During the wind-up phase, the kicking hip begins to flex and continues to flex throughout the remainder of the kicking motion, while the kicking knee begins to flex in preparation for a rapid extension in the following forward swing phase. Although the right knee is still flexing, the quadriceps replace the hamstring as the most active muscle group, presumably to decelerate the knee through eccentric contraction in order to begin the forward swing. The forward swing is a phase of continued hip flexion as well as rapid knee extension and ends with ball contact. The rectus abdominis, as well as the quadriceps of the kicking leg, sees substantial increase in activity during this phase. Follow-through includes the point of ball contact where the knee is still partially flexed to approximately 50 degrees. Follow-through lasts until the knee reaches full extension. During recovery, the right hip reaches full flexion and both feet typically leave the ground [8].

Punt kicking is analogous to a throwing motion with much of the work of the kick performed eccentrically in the early phases of the kick by proximal muscle groups and the momentum transferred to the distal segments of the leg just before ball contact [8]. Through this motion, the kicking limb experiences muscle forces and impact in the absence of weight bearing while the support leg experiences high-grade muscle forces simultaneously [16,38-42]. During the punt, the most active muscle group is the quadriceps of the kicking leg, which acts eccentrically in the wind-up and concentrically in the forward swing. High activity can also be observed in the adductor longus and tensor fascia latae of the kicking leg when imaged with MRI following kicking exercise [14]. The semitendinosus and tensor fascia latae of the support leg also have high activity on MRI [16]. The hamstring of the kicking leg is largely responsible for the concentric initiation of the backswing. The high activities in the kicking leg’s quadriceps, adductors, rectus abdominis, stance leg’s gluteal muscles, and both hamstrings help explain the high rates of muscular injuries in the Australian football league [8]. The activity of muscle groups within the lower extremity is dependent and different based on whether the support or kicking leg is being observed; however, there is little difference between the EMG profiles of the kicking leg for left and right foot dominant kickers [8].

It is also necessary to evaluate the joint kinematics of a punt kick, in addition to the muscle activity. Individual analysis of the joints involved in the punt kick showed that the ankle of the support leg began in dorsiflexion and progressed into plantarflexion. The kicking ankle was not studied. The knee experienced varying degrees of flexion depending on whether the kicking or support leg was observed. The hip experienced flexion throughout the movement, and the pelvis moved from neutral to posterior tilt during the last half of the movement [9]. Greater degrees of hip flexion in both limbs as well as greater degrees of knee flexion in the support limb are associated with increased accuracy of the punt kick [9].

In addition to the described kicking phases, muscle activity, and joint kinematics, there is a need for the kicker to minimize the ground reaction forces of the support leg. Minimizing the ground reaction forces of the support leg is associated with a greater predicted distance when punting [34]. Increased foot speed, shank angular velocity at ball contact, and kicking step length are associated with increased kicking distance [43]. This increased distance is desirable for in-game application of the punt kick.

Place kicking is another in-game application of kicking that is common in rugby and American football. This kick is a method of scoring by either field goal or the point after touchdown in American football and is responsible for 45% of points scored in rugby union [37,44]. In comparison to punting, place-kicking precision and accuracy are more important as it is used in a scoring capacity wherein the ball must pass between two uprights and above a crossbar [45]. The mechanics of the kicking leg during a place kick can be broken into two phases, the late approach phase and the kicking phase. The late approach phase is described as the time prior to contact of the support foot with the ground. The late approach phase can further be broken down by describing the last two steps of the approach. The penultimate or "ghost step" is named for the drifting motion of the player during this initial of the two steps. The kicking step is typically longer and performed at a higher intensity [46]. During the late approach phase, the ankle is in slight plantarflexion, the knee joint is in flexion, and the hip joint is in extension until just before support foot contact. The hip undergoes a large flexor moment just before support foot contact. The kicking phase is the time from contact of the support foot to the point of ball contact. During the kicking phase the ankle is dorsiflexed, and the knee continues to flex for the first half of the kicking phase after which extension was observed until ball contact. Powerful hip flexion continues throughout the kicking phase with a second flexor moment occurring during the last 20% of the kicking phase [47]. Arm motion is also an important contributor to the overall mechanics of the place kick. Greater non-kicking-side arm angular momentum about the global antero-posterior axis is associated with more accurate kickers. Increased longitudinal angular momentum of the non-kicking-side arm is also seen with increased distance demand [48]. This implies that an upper-extremity injury could impact kicking performance of these athletes; however, as these types of injuries are relatively rare for these athletes, rehabilitation specific to upper-extremity injuries is outside the scope of this review [1-3]. The movement patterns observed in the place kick are similar to those previously reported for the in-step kick in soccer but with more emphasis on velocity generation from proximal joints. (Did they also talk about a difference in the arc of motion of hip extension to hip flexion requirements for place kicking?) These differences may be due to the greater accuracy constraints imposed on place kickers and, therefore, the need for a more controlled football contact [47]. 

Need for kicking program for punting and place-kicking athletes

As the American football kicking athlete injury profile is unique to the role, interval programs currently developed that are aimed toward European football athletes or for throwing sports will not have the position-specific modifications and exercises for optimal recovery in an American football kicking athlete. Hip injuries make up the largest proportion of the group’s injuries, while in European football, injuries to the thigh region, including the hamstrings and quadriceps, are the most common [7,33]. There is a need for the development of a separate program for the American football kicking athletes. The interval kicking program developed for return to soccer following lower-extremity injury [7] is an in-depth program tailored to injuries and kicking biomechanics in soccer athletes. A similar program specific to American football kicking athletes and their unique injury profiles and biomechanics would be useful for player rehabilitation and expedited return-to-play. A previous case study of a collegiate American football kicker published a kicking program to be utilized two weeks after beginning physical therapy. The athlete would complete 10-15 kickoffs, 20 field goals at distances of 20-55 yards, and ice [49]. An interval kicking program that more specifically outlines a progression for the athlete to follow when returning to kick would allow for a safer return to kicking by ensuring the athlete does not attempt too heavy of a workload during the earlier phases of rehabilitation.

Although place-kicking and punting biomechanics are similar among all kicking athletes, each individual will likely have developed some unique mechanics that will not be accounted for perfectly by a general interval rehabilitation program. These athletes’ movement patterns should be assessed when rehabilitation begins to properly address their individual needs in addition to the use of the interval programs. 

Limitations

The authors acknowledge limitations to the present work. This review showed a clear lack of sport-specific return-to-kicking interval therapy program for American football, rugby, and Australian rules football athletes. The efficacy of these interval recovery programs that have been used in soccer players and for throwing athletes has not been well studied for their improvement in player health over other recovery programs. To best test the hypothesis that these interval training programs decrease recovery time, improve kicking performance, and assist in player readiness for full-contact play in the injured athletes, future studies should be conducted to compare the athlete progression with various recovery programs implemented under circumstances of similar injury.

The injury studies of the American football kicking athletes used were predominantly from the professional level, limiting the applicability of these studies for the use of rehabilitation of injury in recreational, high school, or collegiate athletes. This may cause a discrepancy due to the physical demands and conditioning requirements required of professional-level kicking athletes, diminishing the effectiveness and application of a rehabilitation program tailored to professionals for players of lower caliber. More rigorous and high-powered studies should investigate this discordance between professional and adolescent, recreational, and collegiate-level players. As such, providers must demonstrate increased clinical vigilance when applying these interval programs developed for professional athletes to others. Furthermore, the average age of professional athletes in American football is far greater than that of adolescent and collegiate leagues, confounding the relationship of injuries per player, as increased age was shown to be in association with increased risk of muscle strain and time to return to play following injury. This may also change the injury profile, as professional-level kicking athletes may experience different injuries during their careers than players in other leagues [1]. Thus, it is of high importance to analyze the specific player’s injury and biomechanics when applying these interval rehabilitation programs. 

## Conclusions

Punting and place kicking are physically demanding activities that are performed in sports where the main injury risk is to the lower extremities such as American football, rugby, and Australian rules football. Interval programs have been developed with the goal of ensuring athlete readiness for return to play in various sports such as baseball, golf, tennis, and soccer. Arundale et al. developed an interval kicking program to be used for the soccer athlete; however, at the time the authors conducted this review of the literature there was no interval kicking program designed to aid the rehabilitation of the punting or place-kicking athlete. Punting and place-kicking have both kinematic and in-game application differences that warrant their own interval kicking program to ensure these athletes can return to play as safely as possible. The purpose of this literature review is to establish the current status of interval kicking programs for the punting and place-kicking athlete. During this review it became apparent that there is a gap in the literature surrounding programs for these athletes. To the authors’ best knowledge, there are no specific interval programs published for the punting and place-kicking athlete. Utilizing an interval kicking program in conjunction with physical therapy could represent a modality of rehabilitation that allows athletes to achieve a safer return to sport and decrease the rate of reinjury. As such, there is a need for the development of interval progression programs for the punting and place-kicking athlete following rehabilitation to ensure physical readiness to return to sport in a state that will minimize risk of repeat injury. Future research should be focused on the development and implementation of such a program with a focus on the ability of players to return to their level of play prior to injury as well as the reducing rates of return injury.
